# Postoperative Fluid Overload is a Useful Predictor of the Short-Term Outcome of Renal Replacement Therapy for Acute Kidney Injury After Cardiac Surgery

**DOI:** 10.1097/MD.0000000000001360

**Published:** 2015-08-21

**Authors:** Jiarui Xu, Bo Shen, Yi Fang, Zhonghua Liu, Jianzhou Zou, Lan Liu, Chunsheng Wang, Xiaoqiang Ding, Jie Teng

**Affiliations:** From the Department of Nephrology, Zhongshan Hospital, Shanghai Medical College, Fudan University (JX, BS, YF, ZL, JZ, XD, JT); Kidney and Dialysis Institute of Shanghai (JX, BS, YF, ZL, JZ, XD, JT); Kidney and Blood Purification Laboratory of Shanghai (JX, BS, ZL, JZ, XD, JT); and Department of Cardiovascular Surgery, Zhongshan Hospital, Shanghai Medical College, Fudan University, Shanghai, China (LL, CW).

## Abstract

To analyze the predictive value of postoperative percent fluid overload (PFO) of renal replacement therapy (RRT) for acute kidney injury (AKI) patients after cardiac surgery.

Data from 280 cardiac surgery patients between 2005 January and 2012 April were collected for retrospective analyses. A receiver operating characteristic (ROC) curve was used to compare the predictive values of cumulative PFO at different times after surgery for 90-day mortality.

The cumulative PFO before RRT initiation was 7.9% ± 7.1% and the median PFO 6.1%. The cumulative PFO before and after RRT initiation in intensive care unit (ICU) was higher in the death group than in the survival group (8.8% ± 7.6% vs 6.1% ± 5.6%, *P* = 0.001; −0.5[−5.6, 5.1]% vs 6.9[2.2, 14.6]%, *P* < 0.001). The cumulative PFO during the whole ICU stay was 14.3% ± 15.8% and the median PFO was 10.7%. The areas under the ROC curves to predict the 90-day mortality by PFO at 24 hours, cumulative PFO before and after RRT initiation, and PFO during the whole ICU stay postoperatively were 0.625, 0.627, 0.731, and 0.752. PFO during the whole ICU stay ≥7.2% was determined as the cut-off point for 90-day mortality prediction with a sensitivity of 77% and a specificity of 64%. Kaplan–Meier survival estimates showed a significant difference in survival among patients with cumulative PFO ≥ 7.2% and PFO < 7.2% after cardiac surgery (log-rank *P* < 0.001).

Postoperative cumulative PFO during the whole ICU stay ≥7.2% would have an adverse effect on 90-day short-term outcome, which may provide a strategy for the volume control of AKI-RRT patients after cardiac surgery.

## INTRODUCTION

Acute kidney injury (AKI) after cardiac surgery is a common complication in intensive care unit (ICU) patients and is associated with high mortality, particularly when renal replacement therapy (RRT) is required.^1^ Fluid overload can readily occur because the cardiac surgery can affect the pumping actions of the heart, leading to postoperative hemodynamic instability.^2^ The condition often remains symptomless for several days until clinical symptoms become apparent, when treatment is almost always too late and ineffective. In recent years, fluid overload has been identified as a new indicator of prognosis for critically ill patients with AKI.^3^ The diagnosis of AKI is typically based on an elevation in serum creatinine (SCr) and blood urea nitrogen levels. However, it has been suggested that waiting for these variable levels to rise as a diagnosis of AKI may delay early intervention for this devastating medical condition. Thus, the prompt management of fluid overload is now considered to be very important during intensive care.^4^ Previously the view was held that fluid accumulation is an important determinant of RRT initiation in critical illness, a symptom that may surpass changes in conventional solute/metabolic parameters.^5^ Many studies have strongly indicated that a percent fluid overload (PFO) that becomes greater than 10% of body weight before the initiation of RRT is associated with a worsening prognosis.^6–8^ Our previous study also confirmed that the 30-day mortality was significantly higher in patients with early postoperative fluid overload.^9^ However, most published studies had one thing in common, namely that they focused on the relationship between the cumulative PFO before the initiation of RRT or early PFO and prognosis.^5,10,11^ It seems that PFO might have been calculated only as indication of RRT initiation, but until now there are no studies about this and few studies have unequivocally established the relationship between PFO after RRT initiation and prognosis. The aim of our study was to determine the relationship between postoperative PFO at different time periods and the overall short-term outcome.

## PATIENTS AND METHODS

### Patients

In this retrospective study, data were collected from 300 AKI patients who received RRT after cardiac surgery in Zhongshan Hospital between January 2005 and April 2012. After excluding 20 patients who were <18 years of age or those who died within 72 hours of admission to the ICU, a cohort of 280 patients were enrolled. According to the AKI network standard,^12^ AKI was defined as SCr absolute value increases of ≥26.4 μmol/L within 48 hours or increases of ≥50% from the standard baseline value, which was the lowest SCr value after admission and before an operation. Diuretics were administered to most patients after their operations and standard urine output was defined as a urine volume of >0.5 mL/kg/hour after >6 hours, when the first dose of furosemide was administered (80 mg), with the highest dose being 240 mg. Fluid intake and output was recorded before surgery and throughout the whole stay of a patient in the ICU. PFO was calculated as the total fluid input (L) − total fluid output (L)/basic weight (kg) × 100%.^13^

Diarrhea dehydration, blood drainage, and hemodialysis dehydration have been included into the daily fluid output. Basic weight was the patient's weight on the day of hospital admission. The indications for RRT initiation were as follows: serum K+ ≥6.0 mmol/L and/or electrocardiogram abnormalities; arterial blood pH ≤7.20; urine output <200 mL/12 hours when the first dose of furosemide was up to 80 mg or the highest dose was up to 240 mg, or anuria; rapidly rising blood urea nitrogen or SCr; refractory fluid overload with pulmonary edema; and severe sepsis with septic shock. The type of RRT was prolonged intermittent RRT which lasted about 8 to 16 hours per session and which were performed almost every day.^14^ The study was approved by the ethics committee of the Zhongshan Hospital. Written informed consent was obtained from all participants.

## METHODS

We performed a receiver operating characteristic (ROC) analysis in order to derive the predictive PFO within 24 hours, PFO before and after RRT initiation and the whole ICU stay for 90-day mortality. Demographic data and perioperative conditions were also recorded. Each patient was transferred to the ICU immediately after his or her operation where hemodynamic parameters such as heart rate and blood pressure were routinely monitored. Acute Physiology and Chronic Health Evaluation (APACHE) II and Sequential Organ Failure Assessment (SOFA) scores were determined within 24 hours postoperatively and renal function was measured daily, while fluid input and output were carefully recorded. Patients with severe AKI received RRT (bedside hemodialysis) when they were in a stable hemodynamic condition without severe sepsis after cardiac surgery. Bedside hemofiltration or extended daily hemodialysis was applied to patients with unstable hemodynamic and high volume hemofiltration with severe sepsis or multiple organ dysfunction syndrome. The primary end point was the 90-day mortality.

### Statistical Analyses

All data were analyzed using SPSS Version 16.0 statistical software (SPSS Inc., Chicago, IL). Histograms were constructed to test for normality of the data distribution. Measurement data with a normal distribution are shown as the mean ± SD, whereas non-normally distributed data are shown as the median with 4 percentile intervals [M (QR)] and count data are described by frequency. Comparisons between groups were performed using a 2 independent sample *t*-test, Wilcoxon rank sum, or χ^2^ tests. The ROC method was used to analyze the predictive value of PFO at different time points after cardiac surgery for 90-day mortality. The Hosmer–Lemeshow goodness of fit test was performed for the calibration curve. A bilateral *P* value <0.05 was considered to be statistically significant.

## RESULTS

### Baseline Characteristics

The cohort of 280 patients who received RRT after cardiac surgery was comprised of 200 males and 80 females with a mean age of 56 ± 14 years (range 18–83). The average hospital stay was 36 ± 35 days (range 3–210) and the average stay in ICU was 20 ± 24 days (range 3–173). The 90-day mortality was 65% (n = 182). Patients were divided into death and survival groups, based on the 90-day survival. The age in the death group was significantly higher than in the survival group, but the sex, preoperative heart and renal functions, comorbidity of diabetes and hypertension, cardiopulmonary bypass time, aortic cross-clamp time, and APACHE II and SOFA scores within 24 hours postoperatively were not significantly different between the 2 groups (Table 1). There was no significant difference of medications used before surgery. After surgery, adrenaline and noradrenaline were more applied in the death group while digoxin and warfarin were more used in the survival group. PFOs, at different time periods including the whole ICU stay after operation, in the death group were significantly higher than in the survival group. The durations between admission to the ICU and RRT initiation were not significantly different between the 2 groups (*P* > 0.05).

**TABLE 1:**
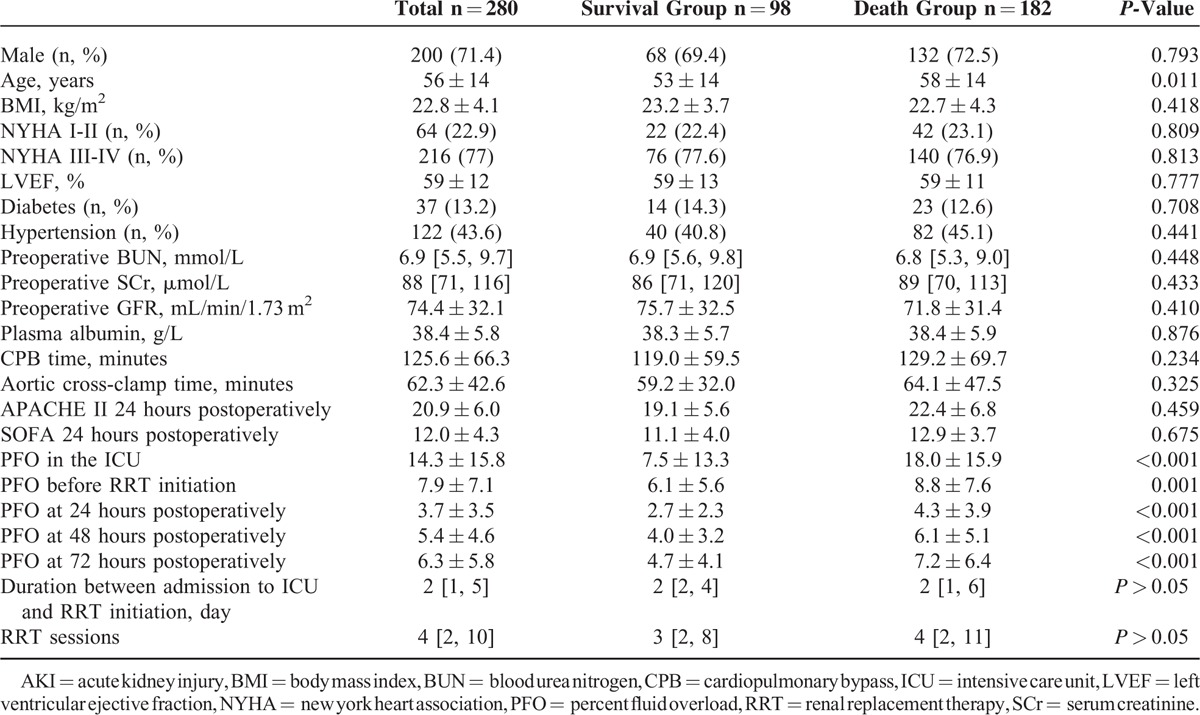
Comparison of Clinical Data of AKI-RRT Patients After Cardiac Surgery

### PFO Data

The mean PFO before RRT initiation was 7.9% ± 7.1% and the median PFO was 6.1%. The cumulative PFO before RRT initiation was higher in the death group than in the survival group (8.8% ± 7.6% vs 6.1% ± 5.6%, *P* = 0.001). The mortality of patients with a PFO ≥10% before the initiation of RRT was significantly higher than those with a PFO <10% (76.0% vs 59.6%, *P* < 0.001). The cumulative PFO after the initiation of RRT was significantly higher in the death group compared with the survival group (−0.5 [−5.6, 5.1]% vs 6.9 [2.2, 14.6]%, *P* < 0.001). The mean PFO during the whole ICU stay was 14.3% ± 15.8% and the median PFO was 10.7%. The mean PFO during the whole ICU stay was higher in the death group than in the survival group (18.0% ± 15.9% vs 7.5% ± 13.3%, *P* < 0.001).

The cumulative PFO 24, 48, and 72 hours postoperatively increased from 3.7% ± 3.5%, 5.4% ± 4.6%, and 6.3% ± 5.8%, respectively. The mean PFO within 24 hours was higher in the death group than in the survival group (4.3% ± 3.9% vs 2.7% ± 2.3%, *P* < 0.001).

### Predictive Value of Postoperative PFO in Different Time Periods for 90-day Mortality

**FIGURE 1:**
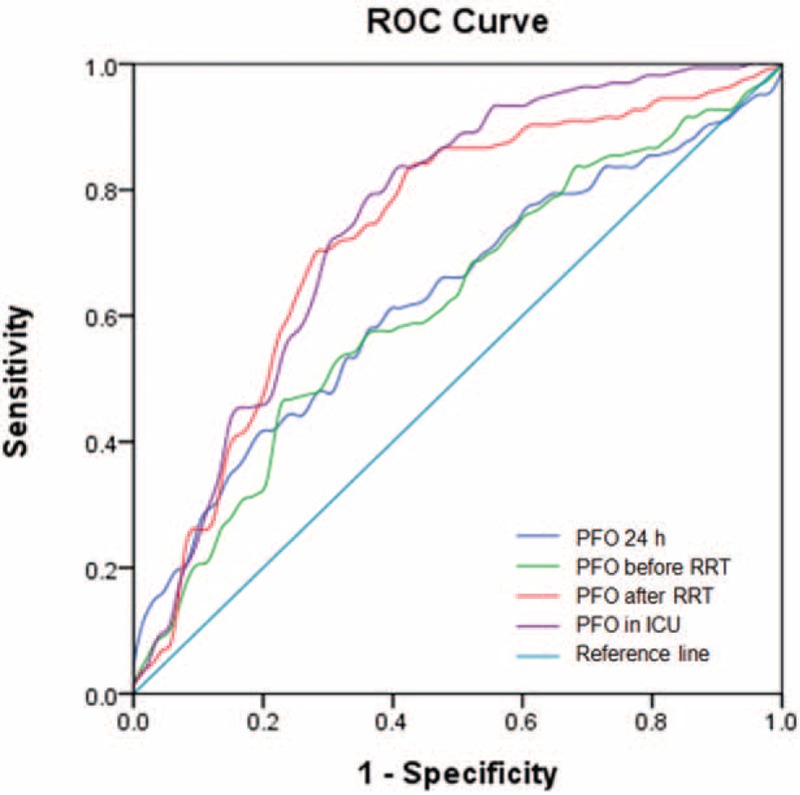
The area under the ROC curve to predict 90-day mortality by PFO within 24 hours, PFO before and after RRT initiation. PFOs during the whole ICU stay postoperatively were 0.625 (95% CI: 0.557–0.693, *P* < 0.01), 0.627 (95% CI: 0.557–0.697, *P* < 0.01), 0.731 (95% CI: 0.662–0.798, *P* < 0.001), and 0.752 (95% CI: 0.687–0.818, *P* < 0.001), respectively. CI = confidence interval, ICU =  intensive care unit, PFO = percent fluid overload, ROC = receiver operating characteristic, RRT = renal replacement therapy.

The areas under the ROC curves which were used to predict the 90-day mortality by PFO within 24 hours, PFO before and after RRT initiation, and PFO during the whole ICU stay postoperatively were 0.625 (95% confidence interval [CI]: 0.557–0.693, *P* < 0.01), 0.627 (95% CI: 0.557–0.697, *P* < 0.01), 0.731 (95% CI: 0.662–0.798, *P* < 0.001), and 0.752 (95% CI: 0.687–0.818, *P* < 0.001), respectively (Figure 1). An area under the ROC curves >0.7 has usually clinically importance, but it was apparent that only the PFO during the whole ICU stay period postoperatively had a good predictive value. We further calculated the cut-off point and determined a PFO during the whole ICU stay ≥7.2% was the best critical value, with a sensitivity of 77% and a specificity of 64% for the prediction of 90-day mortality.

**FIGURE 2:**
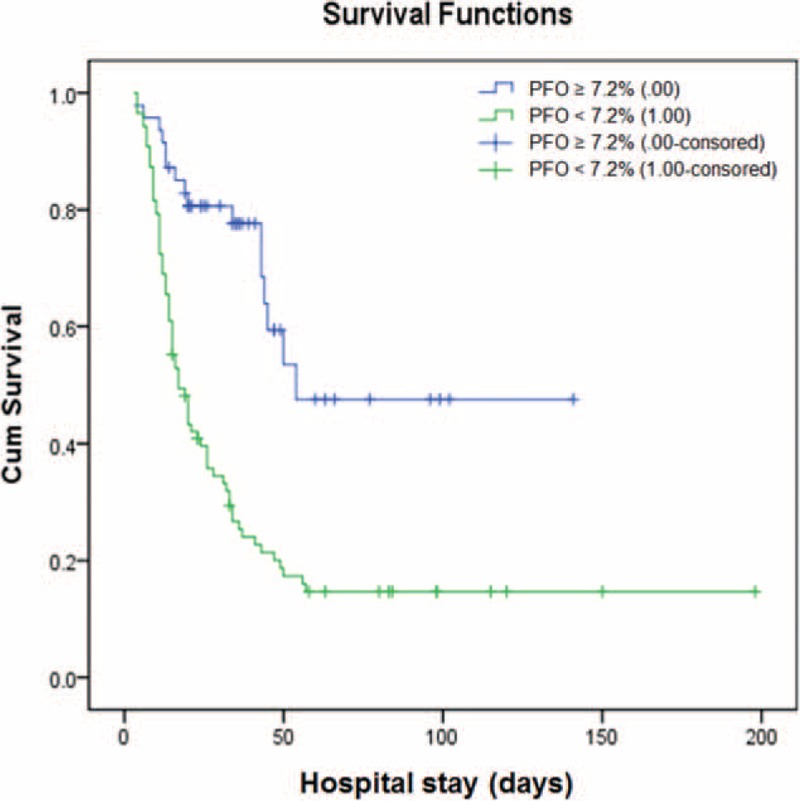
Kaplan–Meier survival estimates by fluid overload status. There was a significant difference in survival rates among patients with a cumulative percent fluid overload (PFO) ≥ 7.2% and a PFO < 7.2% after surgery (log-rank *P* < 0.001).

Kaplan–Meier survival estimates showed that there was a significant difference in survival among patients with a cumulative PFO ≥ 7.2% and a PFO < 7.2% after cardiac surgery (log-rank *P* < 0.001) (Figure 2).

## DISCUSSION

Our study has shown that a high PFO during different time periods after cardiac surgery might beside other factors lead to a worsened prognosis. The Sepsis Occurrence in Acutely Ill Patients study had already confirmed that a positive fluid balance was an independent factor associated with increased 60-day mortality^15^ and a previous study of our research group has also confirmed that fluid overload during the whole ICU stay was a risk factor for 30-day mortality.^16^ The ROC curves of PFO within 24 hours, PFO before and after RRT initiation, and PFO during the whole ICU stay postoperatively, were employed to predict the 90-day mortality. The results suggest that the area under the ROC for the whole ICU stay period was relatively larger (up to 0.752), with a cut-off point of 7.2%.

The PFO before RRT initiation has always been considered to be an important predictor of mortality. Much data supporting this view have come from retrospective pediatric studies in dialyzed patients. Goldstein et al^17^ first defined the PFO as the difference of fluid input and output volume (L) / weight (kg) ∗100% and found that PFO at the initial time of continuous venovenous hemofiltration in the death group of children was much higher than that in the survival group. Gillespie et al^13^ used the above algorithm and set a median PFO of 10% as the cut-off point, and reported that the death risk of patients with PFO over 10% (high load group) at RRT initiation was 3 times (OR = 3.02) higher than those with a PFO less than 10% (low load group). They concluded that starting RRT when the PFO was over 10% would have an adverse effect on the prognosis.

Some authors held different opinions about the definition of the cut-off point of fluid overload. Bagshaw et al^5^ conducted a prospective multicenter study for severe AKI patients and reported that fluid balance at RRT initiation was 3000 mL/24 hours, and 92.2% of patients were in a positive fluid balance at RRT initiation, 38.3% had a %FO ≥ 5%, and 18.1% had a %FO ≥ 10%. The mortality rose when the PFO was greater than 5% or the positive balance of fluid volume was more than 3 L/24 hours at RRT initiation.

PFO before RRT initiation is indeed a key indicator. PFO is most likely to occur in critically ill patients after cardiac surgery because fluid input would obviously be increased due to the low cardiac output. Thus, the higher cumulative liquid load before RRT initiation would indicate a severe illness status, and this may account for the higher mortality in patients with a higher fluid load before RRT initiation.^6–8,18^ However, in the present study, the area under the ROC curve for PFO before RRT initiation was only 0.627, which is far lower than the area under the ROC curve for PFO (area 0.752) during the whole ICU stay. We believe that PFO before RRT initiation, which seemingly did not incorporate the effect of subsequent RRT on prognosis, may not be a good indicator of outcome. The prognosis may be improved if the PFO is controlled after RRT treatment, so that the PFO during the whole ICU stay may well be a better predictor of the outcome.

A multicenter prospective study in 2009 [The Program to Improve Care in Acute Renal Disease (PICARD)] investigated 618 critically ill patients with AKI.^6^ Our study partly confirmed the PICARD findings and the fluid load in that study included all liquid load 3 days before AKI occurred to discharge. They found in patients requiring RRT, that survivors had a significantly lower PFO at dialysis cessation compared with nonsurvivors (13.0% vs 22.1%; *P* = 0.002), and patients with fluid overload at RRT initiation who ended RRT without fluid overload (PFO < 10%) were less likely to die than those who still had fluid overload (PFO ≥ 10%) at the cessation of RRT. Patients who continued to have fluid accumulation throughout their hospital stay were more likely to die, suggesting a cumulative effect of fluid overload on mortality. It was clear that mortality was lower when fluid overload was corrected by dialysis. Furthermore, it was not appropriate to use 10% PFO as the reference standard to define fluid overload in our patients. In contrast to the definition of fluid overload as 10% PFO provided by Gillespie et al^13^ (2004) using the median of the cumulative fluid load based on a population of children, in our study the median PFO before RRT was only 6.1%.

Our study provides a significant breakthrough method, by using fluid overload throughout a patient's entire stay in ICU, to predict outcomes for Chinese patients. However, it is important to note that our research was as a single-center retrospective study with a relatively small sample size and some bias may have existed. In our study, the best cut-off value of PFO during the ICU-stay period was 7.2%, which refers to the sum of the PFO before and after RRT. We advise that PFO should be monitored and controlled strictly after RRT treatment and that PFO after RRT may be of greater significance for the improvement of the prognosis. The study was conducted over a long period and control of water balance could have progressed during the study in parallel with the progress of other treatments. In fact, the RRT machine (Aquarius CRRT equipment) has not been changed during these years, since the machine can meet almost all the treatment requirements, is easy to move and to handle and we did not think that changing essential equipment is favorable for nurses and engineers. The dose and modality of RRT has also not been essentially changed, but during the recent years we have become aware of the trend that PFO after RRT might be of importance and the timing of RRT initiation has been shifted to earlier than before, while the duration of each RRT session has been prolonged. Further randomized studies about fluid balance control strategies comparing 2 fluid balance control strategies should be conducted to further confirm the link between fluid overload and mortality. Nevertheless, we think that the results presented in this article provide guidance for the volume control of critically ill AKI patients.

## CONCLUSION

PFO measured throughout the whole ICU stay period postoperatively for AKI-RRT patients is a good predictor of the short-term outcome. Fluid overload ≥7.2% after cardiac surgery had a serious adverse effect on prognosis. Therefore, PFO measurements may provide a useful new strategy for volume control in critically ill patients with AKI after cardiac surgery.
